# Melon GliSODin^®^ Prevents Diet-Induced NASH Onset by Reducing Fat Synthesis and Improving Liver Function

**DOI:** 10.3390/nu11081779

**Published:** 2019-08-01

**Authors:** Anna Nakamura, Naho Kitamura, Yoko Yokoyama, Sena Uchida, Kayo Kumadaki, Kazuo Tsubota, Mitsuhiro Watanabe

**Affiliations:** 1 Systems Biology Program, Graduate School of Media and Governance, Keio University, Kanagawa 252-0882, Japan; 2 Health Science Laboratory, Keio Research Institute at SFC, Kanagawa 252-0882, Japan; 3 Department of Ophthalmology, Keio University School of Medicine, Tokyo 160-8582, Japan; 4 Department of Environment and Information Studies, Keio University, Kanagawa 252-0882, Japan

**Keywords:** non-alcoholic steatohepatitis, non-alcoholic fatty liver disease, oxidative stress, liver disease, natural products

## Abstract

A high-calorie diet causes fat accumulation and oxidative stress in the liver, leading to fatty liver and eventually non-alcoholic steatohepatitis (NASH). Melon GliSODin^®^ is used as a nutritional supplement because of its antioxidant activity. This study aimed to assess the antioxidant activity of Melon GliSODin^®^ and its effectiveness in preventing NASH, which primarily results from oxidative stress. Furthermore, we verified the protective effect of Melon GliSODin^®^ by administering it to a mouse model of diet-induced NASH. Melon GliSODin^®^ suppressed liver fibrosis and fat accumulation, which is characteristic of the NASH phenotype. Gene expression analysis confirmed the suppression of fat synthesis and activation of antioxidative mechanisms. These results show that Melon GliSODin^®^ mitigates NASH onset at the molecular level, suggesting its potential application as a NASH preventive agent.

## 1. Introduction

Antioxidants quench reactive oxygen species [1]; however, their levels decrease with age, causing a corresponding increase in oxidative stress [2,3,4]. Oxidation leads to the production of free radicals leading to chain reactions potentially causing damage to living organisms. It is, therefore, becoming progressively more important to consume antioxidants with food or as dietary supplements. In recent years antioxidants have been referred to as a seventh category of nutrients besides proteins, carbohydrates, lipids, vitamins, minerals, and dietary fiber. Accumulation of oxidative stress is considered to contribute to the development of obesity and other lifestyle-related diseases [5].

Non-alcoholic fatty liver disease (NAFLD) usually occurs in individuals with obesity and insulin resistance [6], but not in those with alcoholic liver injury. NAFLD is currently estimated to affect 20–30% of the total population and is a major public health concern worldwide, owing to the increased worldwide incidence of obesity [7,8]. Non-alcoholic steatohepatitis (NASH) is a progressive form of NAFLD characterized by liver steatosis and inflammation [9]. Numerous studies have reported that NASH leads to progressive fibrosis and eventually cirrhosis or liver cancers. However, no effective therapy is currently available and the mechanism is unclear [10].

Two principal theories have been proposed to explain the origin of NASH [11]. The first is the onset of insulin resistance [12] and the second is an increase in oxidative stress. However, while the etiology of NASH comprises numerous unclear aspects [13], it is important to establish effective preventive strategies and treatments based on the current understanding of the disease.

Since oxidative stress contributes to NASH progression [14], factors reducing its severity should help prevent NASH. Oxidative stress is caused by oxidative reactions affecting cells containing high levels of reactive oxygen species (ROS) [15]. ROS are usually detoxified by antioxidant mechanisms such as those involving the enzymes superoxide dismutase (SOD) and catalase. SOD is effective enzyme that eliminates oxidative stress [16]. However, the pathological condition of the liver in NASH is characterized by increased ROS production, exceeding the protective capacity of the antioxidant systems and inducing oxidative stress [17]. Consequently, to suppress ROS generation to prevent the onset of NASH, resulting from obesity and lifestyle-related diseases, the antioxidant capacity of liver cells needs to be improved.

Melon GliSODin^®^ is a supplement produced from original vegetable formula made from a SOD-rich melon extract (*Cucumis melo* LC). Melon GliSODin^®^ is coated with a Gliadin molecule, a protein from wheat, which degrades in the stomach and allows it to reach the intestine [5]. These are antioxidant rich and three to four times more resistant to decay than other melons; moreover, they contain up to seven times the amount of SOD present in other melons. Several studies have shown that this enzyme is the most potent molecule for removing ROS species.

Several studies using Melon GliSODin^®^ have been conducted using several animal models. Particularly, in animal studies, Melon GliSODin^®^ increases the activity of antioxidant enzymes in circulating blood [18] and to decrease the production of inflammatory cytokines [19]. Although Melon GliSODin^®^ exerts antioxidant and anti-inflammatory effects, no studies have attempted to verify these effects in NASH.

Therefore, based on the information presented above, we hypothesized that the antioxidant action of Melon GliSODin^®^ might reduce oxidative stress in the liver and prevent the onset of NASH. In this study, we investigated the effect of Melon GliSODin^®^ in dietary-induced NASH model mice.

## 2. Materials and Methods 

### 2.1. Melon Extract

Melon GliSODin^®^ was obtained from the Nutrition Act Co., Ltd., Chūō-ku, Japan.

Animal Studies: all animal experiments were performed in accordance with the standards set forth in the Guidelines for the Use and Care of Laboratory Animals at Keio University, Japan. The protocols were approved by the Institute for Experimental Animals of Keio University. Male C57BL/6J mice, five weeks of age (*n* = 7), were obtained from Japan SLC Inc. All mice were maintained in a temperature-controlled (23 °C) facility on a 12 h light/dark cycle and were provided ad libitum access to food and water over a period of 21 weeks prior to euthanasia and body weights were recorded regularly as presented under results. Mice were divided into three experimental groups (*n* = 7/group). Each group received a normal diet (Control), high-fat and high-cholesterol diet (HC) and high-fat and high-cholesterol diet added 1% w/w Melon GliSODin^®^ (MEL). Animal feeds were obtained from Research Diets Inc. (New Brunswick, NJ, USA). The high-fat and high-cholesterol diet (D09100301) comprised 40 kcal% fat (Mostly Primex), 20 kcal% fructose and 2% cholesterol. The matched control diet (D09100304) comprised 10 kcal% fat with no fructose or cholesterol. All mice were fasted for 6 h before harvesting blood and tissues for analysis, including RNA isolation and histology.

### 2.2. Histology and Staining Analysis

Liver tissues were harvested and immediately fixed with 10% neutral buffered formalin (Sigma) and Bouin’s fixative and paraffin-embedded blocks were prepared. Haematoxylin and eosin (H&E) staining, Oil Red O staining, Sirius red staining, Masson’s trichrome staining and Azan staining were performed for the paraffin-embedded tissue sections. 

### 2.3. Lipid Parameters: Blood Chemistry and Liver Tissue Analysis

Blood samples were collected upon euthanasia and plasma was harvested via centrifugation. Liver extracts were used to quantify cholesterol, non-esterified fatty acid (NEFA), and triglyceride (TG) levels and prepared using the classical Folch method as previously described [20].

Plasma and liver total cholesterol, free cholesterol, NEFA, and TG were determined using enzymatic assay kits (LaboAssayTM series, FUJIFILM Wako Pure Chemical Corporation, Osaka, Japan). Alanine aminotransferase (ALT) and aspartate aminotransferase (AST) activities were determined using enzymatic assay kits (Alanine Aminotransferase Activity Assay Kit and Aspartate Aminotransferase Activity Assay Kit, Bio Vision Inc., Milpitas, California, USA) in accordance with the manufacturer’s instructions.

### 2.4. mRNA Expression Analysis via Quantitative RT-PCR

Total RNA was extracted from tissue samples, using the RNeasy Mini Kit (Qiagen, Hilden, Germany). cDNA was synthesized from total RNA, using the Prime Script RT Reagent Kit. Expression levels of antioxidant status biomarker proteins were analyzed using cDNA synthesized from total mRNA using real-time PCR. Primer sequences are provided in Table 1.

### 2.5. 2-Thiobarbituric Acid Reactive Substances (TBARS) Measurement 

Individual livers were homogenized in Tissue Protein Extraction Reagent (T-PER), using a Polytron tissue grinder. After centrifugation of the homogenates, the supernatant was harvested and TBARS levels were determined using the TBARS Assay Kit (Cayman Chemical Company, Ann Arbor, MI, USA).

### 2.6. Statistical Analysis

Continuous variables were reported as mean ± standard error of the mean (SEM) values. Statistical analysis was performed using R (version 3.4.0, The R Project for Statistical Computing, St. Louis, MO, USA). Normally distributed data were analysed using one-way analysis of variance (ANOVA) with Bonferroni’s post hoc test. Statistical significance is displayed as *p* < 0.05 (^#^/*), *p* < 0.01 (^##^/**) or *p* < 0.001 (^###^/^***^). ^#^Significant differences HC versus Control, *Significant differences HC versus MEL.

## 3. Results

### 3.1. Melon GliSODin^®^ Prevents Lipid Accumulation in the Mouse Model of Diet-Induced NASH

**Figure 1:**
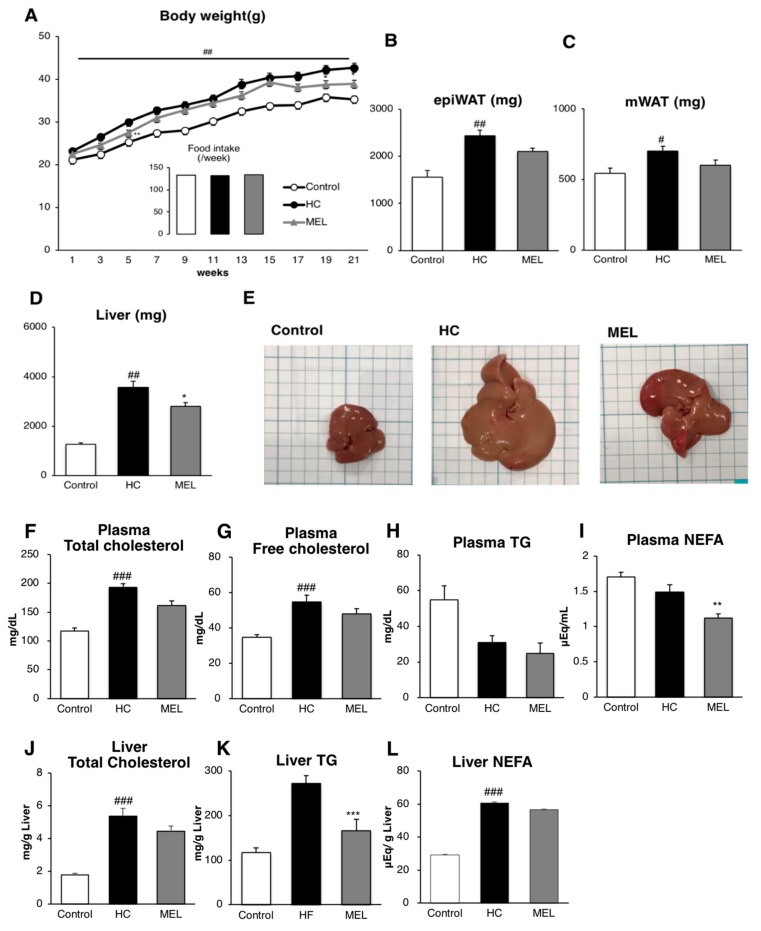
Melon GliSODin^®^ prevents diet-induced non-alcoholic steatohepatitis. Control diet (Control), high-fat and high-cholesterol diet (HC), and high-fat, high-cholesterol + 1% Melon GliSODin^®^ diet (MEL). (**A**) Evolution of body weight gain and food intake with different diets. (**B**) Weight of epidydimal white adipose tissue (WAT). (**C**) Weight of mesenteric WAT. (**D**) Weight of the liver. (**E**) Liver photographs of the control, HC, and MEL groups. (**F**) Plasma lipid parameters, (**G**) total cholesterol, (**H**) triglycerides (TG), and (**I**) non-esterified fatty acid (NEFA). (**J**) Liver total cholesterol, (**K**) TG, and (**L**) NEFA. Data are shown as mean ± SEM values. *n* = 7 mice per group. Statistical analysis was performed using one-way ANOVA followed by the Bonferroni’s post hoc test. ^#^/^*^ *p* < 0.05; ^##^/^**^ *p* < 0.01, ^###^/^***^ *p* < 0.001 versus mice fed HC diet. (^#^Significant differences HC versus Control, *Significant differences HC versus MEL).

We first evaluated metabolic changes induced by Melon GliSODin^®^. Body weight measurements indicated that weight gain was suppressed in the MEL group as compared to the HC group without any change in food intake (Figure 1A).

Liver hypertrophy was clearly suppressed in the Melon GliSODin^®^-administered group rather than in the HC group, as indicated by both liver weights and via visual inspection of the organs, as shown in post-dissection photographs (Figure 1D,E). Furthermore, the weights of epididymal and mesenteric adipose tissues were reduced in comparison with the HC group (Figure 1B,C).

We analyzed lipid parameters in plasma samples harvested during euthanasia and the results show that total cholesterol in the MEL group decreased significantly and other parameters also tended to decline (Figure 1F–I) relative to the HC group.

TGs in the liver were significantly reduced in comparison with the HC group (Figure 1K) with the other parameters showing a similar reducing trend (Figure 1F–I,J,L). These results suggest that Melon GliSODin^®^ may reduce lipid accumulation, which causes NASH.

### 3.2. Melon GliSODin^®^ Prevents Liver Fibrosis in A Mouse Model Of Hc-Induced NASH

**Figure 2:**
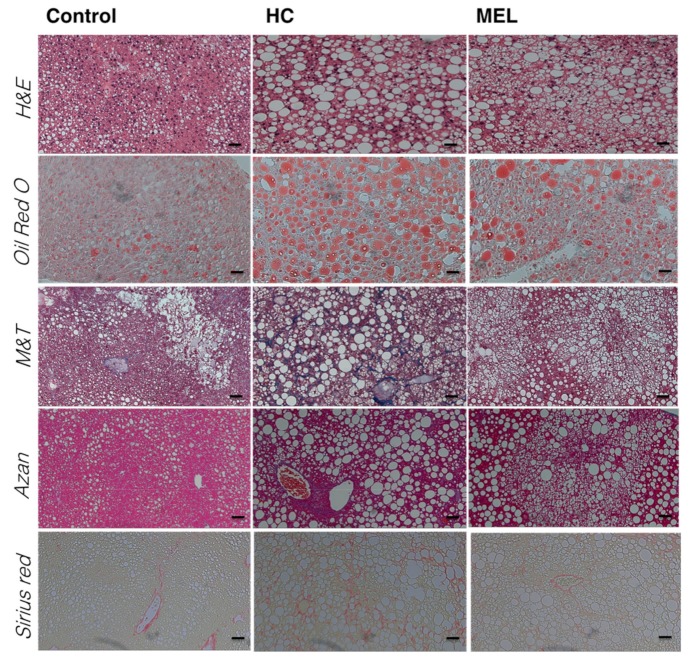
Histological analysis of the liver of the mouse model of a high-fat diet-induced non-alcoholic hepatic steatosis. Scale bar, 50 μm. Animals were treated with three different diets: control diet (Control), high-fat and high-cholesterol diet (HC) and high-fat, high-cholesterol + 1% Melon GliSODin^®^ diet (MEL). Haematoxylin and eosin (H&E) staining; Oil Red O staining; Masson’s trichrome staining; Azan staining; Sirius Red staining.

Histological analysis of livers (Figure 2) revealed that while an increase in lipid droplets was confirmed in the liver of the HC group, lipid droplets were suppressed upon Melon GliSODin^®^ administration (H&E staining and Oil Red O staining).

Masson’s trichrome staining (M&T) staining, Azan staining and Sirius Red staining were carried out for pathological analysis of liver fibrosis [21], and HC group samples displayed clear liver fibrosis. In contrast, the MEL group did not show recognizable liver fibrosis upon histological analysis.

These results indicate that Melon GliSODin^®^ effectively suppresses liver fibrosis.

### 3.3. Melon GliSODin^®^ Attenuated Liver Inflammation and Fibrosis at the Molecular Level

**Figure 3:**
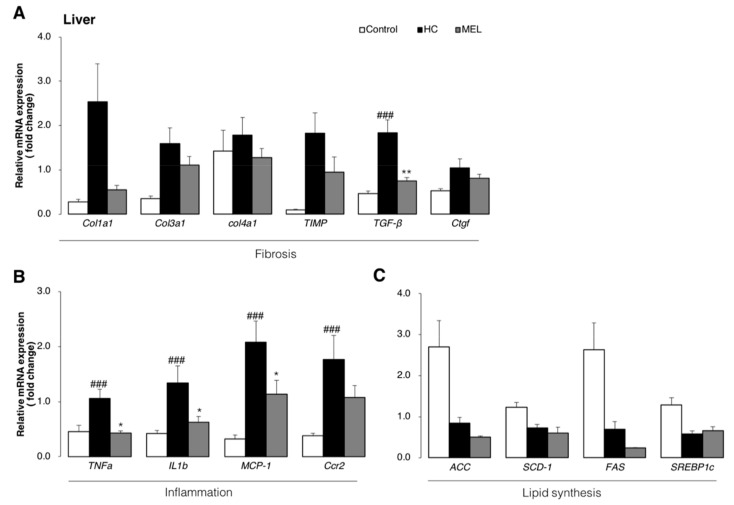
Melon GliSODin^®^ prevents non-alcoholic hepatic steatosis progression at the level of gene expression. Control diet (Control), high-fat and high-cholesterol diet (HC) and high-fat, high-cholesterol + 1% Melon GliSODin^®^ diet (MEL). (**A**) Liver mRNA analysis for fibrosis formation. (**B**) Liver mRNA analysis related to inflammation. (**C**) Liver mRNA analysis related to lipid synthesis. Data are presented as mean ± SEM values. *n* = 7 mice per group. Statistical analysis was performed using one-way ANOVA followed by the Bonferroni’s post hoc test. ^#^/^*^p < 0.05; ^##^/^**^p < 0.01, ^###^/^***^p < 0.001 versus mice in HC group. (^#^Significant differences HC versus Control, *Significant differences HC versus MEL).

To confirm whether the suppression of liver fibrosis by Melon GliSODin^®^ is regulated at the level of gene expression, the expression of collagen type 1 alpha (col1a1), collagen type 3 alpha 1 (col3a1) (fibrosis markers) and transforming growth factor beta (TGF-β) (an upstream regulator of col1a1 and col3a1) were determined [21]. All three factors were suppressed in the MEL group in comparison with the HC group (Figure 3A). We, therefore, conclude that these changes at the level of gene expression may help prevent the onset of NASH.

Furthermore, inflammatory marker genes including tumor necrosis factor-alpha (TNF-α) and interleukin 1-beta (IL-1β) were downregulated by Melon GliSODin^®^ administration relative to the HC group, almost approaching baseline levels in some cases (Figure 3B).

In addition, we focused on lipid accumulation pathways that cause inflammation and evaluated genes involved in lipid synthesis. We measured the expression of the transcriptional regulatory factor sterol regulatory element-binding protein 1c (SREBP1c), which regulates fatty acid synthesis genes including stearoyl-CoA desaturase 1 (SCD1), acetyl CoA carboxylase (ACC) and fatty acid synthase (FAS). In comparison with the HC group and MEL group, these genes tend to be downregulated upon Melon GliSODin^®^ administration (Figure 3C). It is, therefore, considered that reduction of fatty acid synthesis in the liver could attenuate inflammation and oxidative stress.

### 3.4. Melon GliSODin^®^ Boosts Antioxidation Defence Systems in the Hc Diet Loaded Liver.

**Figure 4:**
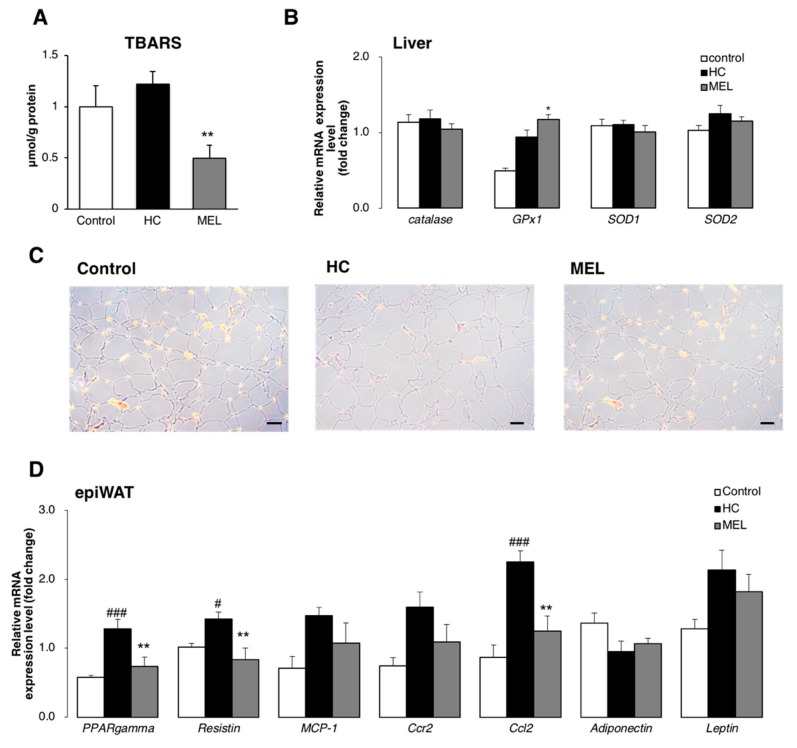
Melon GliSODin^®^ reduces oxidative stress. Control diet (Control), high-fat and high-cholesterol diet (HC) and high-fat, high-cholesterol + 1% Melon GliSODin^®^ diet (MEL). (**A**) Levels of oxidative stress marker TBARS in the liver. (**B**) mRNA analysis of liver antioxidants (**C**) H&E staining of epidydimal adipose tissue. Scale bar, 50 μm. (**D**) mRNA analysis in WAT. Data are shown as mean ± SEM. *n* = 7 mice per group. Statistical analysis was performed using one-way ANOVA followed by the Bonferroni’s post hoc test. ^#^/^*^p < 0.05; ^##^/^**^p < 0.01, ^###^/^***^p < 0.001 versus mice fed a HC diet. (^#^Significant differences HC versus Control, *Significant differences HC versus MEL).

The onset of inflammation and fibrosis in the liver are closely associated with an increase in cellular oxidative stress. We measured TBARS levels in the liver, since this is widely considered a measure of oxidative stress [22]. As shown in Figure 3, fibrosis and inflammation were suppressed upon Melon GliSODin^®^ administration. TBARS values in the MEL group were significantly lower than those in the HC group (Figure 4A) and indeed lower than those in the control group.

Two possible mechanisms or pathways leading to the reduction of oxidative stress have been postulated; the first involves the activation of antioxidant enzymes and the second involves the improvement of mitochondrial function, which directly reduces ROS production.

We quantified the expression of antioxidant genes SOD1 and SOD2, catalase, and glutathione peroxidase (GPx1). The expression of GPx1, which quenches H_2_O_2_ [16], was quantified in the MEL group (Figure 4B). In contrast, no significant differences in SOD or catalase expression were observed in the three experimental groups.

These results confirm that Melon GliSODin^®^ effectively reduces oxidative stress in the liver by increasing the activity of enzymes that quench H_2_O_2_.

Since mitochondria are directly involved in ROS production, we also quantified the expression levels of genes that are markers of mitochondrial function (ND1, 16S; data not shown). However, no significant changes were observed in these factors.

### 3.5. Melon GliSODin^®^ Reduces the Size of Adipocytes and Suppresses the Flux of Free Fatty Acids (FFA) in the Liver

Recent studies have reported that white fat cells, which are major fatty acid-producing tissues, increase systemic FFA levels, an effect associated with the pathogenesis of NASH [23,24].

**Figure 5:**
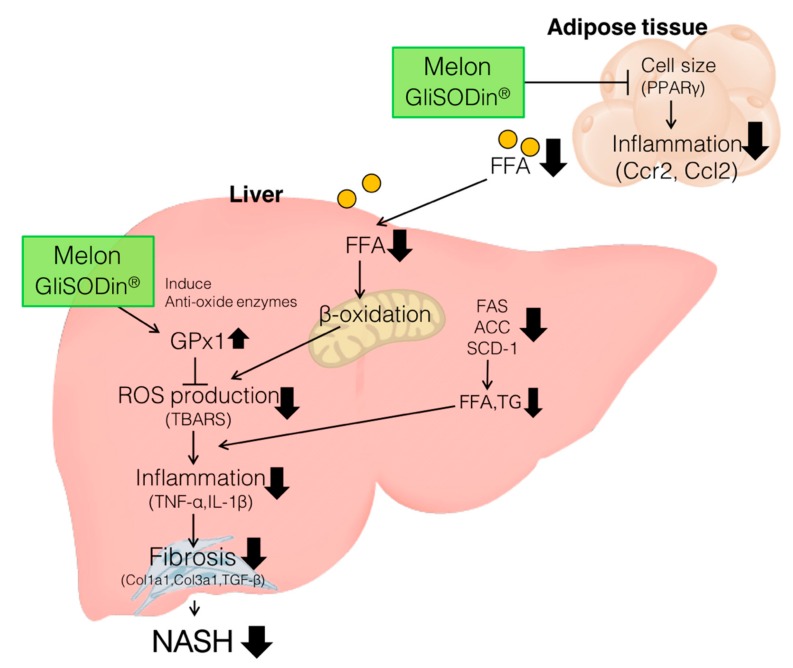
Suggested mechanism for the prevention of NASH by Melon GliSODin^®^.

H&E staining of adipocytes confirmed the reduction in adipocyte size in the MEL group relative to the HC group (Figure 5).

In addition, the peroxisome proliferator-activated receptor gamma (PPAR-γ or PPAR gamma), which regulates adipocyte differentiation at the level of gene expression was inhibited in the MEL group. Leptin and resistin levels were suppressed in the MEL group. Adipocyte macrophage markers, including inflammatory cytokines, C-C motif chemokine 2 (Ccl2), monocyte chemoattractant protein 1 (MCP-1) and C-C Chemokine Receptor Type 2 (Ccr2), were downregulated in the MEL group in comparison with the HC group (Figure 4D). In contrast, adiponectin was slightly upregulated.

These results suggest that the reduced size of fat cells and suppression of inflammation in adipocytes reduced the production and secretion of FFA, and consequently the flux of fatty acids reaching the liver. These results suggest that fatty acid beta-oxidation in the liver would be reduced, thus reducing ROS production.

Melon GliSODin^®^ induces antioxidant enzymes and suppresses ROS levels in the liver. In adipose tissue, a reduction in the size of miniaturized fat cells lowers and reduces the influx of FFA in the liver. The reduction of FFA in the liver suppressed β-oxidation, which potentially reduces ROS production. Furthermore, fatty acid synthesis is potentially suppressed by Melon GliSODin^®^ and thus prevents fat accumulation and inflammation in the liver.

## 4. Discussion

ROS accumulation causes oxidative stress, causes inflammation, apoptosis and dysfunction in other liver cells and is involved in NASH onset.

Under normal conditions, ROS is detoxified by antioxidant enzymes to prevent the accumulation of oxidative stress. However, under fat and cholesterol-rich conditions induced by an HC diet, numerous ROS are produced during β-oxidation of fatty acids (which cannot be processed by antioxidant enzymes), which is caused by lipid peroxidation during oxidative stress and leads to inflammation and cellular damage and is involved in NASH progression [25,26]. Herein, mice fed with an HC diet displayed ROS accumulation along with the accumulation of lipid peroxide marker TBARS in the liver.

In contrast, Melon GliSODin^®^ induces antioxidant enzymes that eliminates ROS and prevents the accumulation of lipid peroxide, which is an indicator of oxidative stress. We initially expected that the antioxidative enzyme SOD would be upregulated in the liver because Melon GliSODin^®^ contains a large amount of SOD. However, neither SOD1 nor SOD2 were upregulated, probably because Melon GliSODin^®^ functions in the same manner as SOD to activate the active oxygen scavenging pathway without upregulating SOD itself and consequently upregulates downstream glutathione peroxidase (Gpx1). Melon GliSODin^®^ administration upregulates downstream antioxidant enzymes in the relevant antioxidant mechanism involving SOD. However, this was investigated only at the mRNA expression level in this study, thus limiting any robust conclusions regarding this pathway.

Furthermore, we clarified that Melon GliSODin^®^ alters the ROS production pathway. Excess fatty acid influx enhances β-oxidation in these organelles and promotes ROS production [27]. Fat cell hypertrophy generated by the accumulation of neutral fat causes inflammation in adipose tissue, and these adipocytes release inflammatory adipocytokines [28,29], such as leptin, TNF-α and free fatty acids. Furthermore, inflammatory adipocytokine induces macrophages in adipose tissue and enhances inflammation in adipose tissue.

Free fatty acids secreted from adipocytes enter the liver [30]. Therein, excess free fatty acids are β-oxidized in the mitochondria, and ROS production occurs at excessive levels, which cannot be processed by the antioxidative system. This results in ROS accumulation [26].

Furthermore, excess fatty acids over the capacity of mitochondrial processing is undergo β-oxidation in peroxisomes. Unlike β-oxidation in peroxisomes, which involves different acyl-CoAs from those in mitochondrial β-oxidation, which is different from the reaction by hydrogenase, the oxidation reaction of acyl-CoA directly produces H_2_O_2_, thus inducing oxidative stress [25]. Our results indicate that Melon GliSODin^®^ suppresses adipocyte hypertrophy and downregulates inflammation-related factors. Consequently, free fatty acids secreted from adipocytes are metabolized in the liver and downregulated during oxidative stress.

Furthermore, we considered the possibility that Melon GliSODin^®^ improves mitochondrial function. Mitochondrial dysfunction is a factor of NASH [31]. On further evaluation, no change was observed in the amount of mitochondrial DNA (data not shown).

These results indicate that Melon GliSODin^®^ potentially reduces β-oxidation in the liver and suppresses ROS production by inhibiting FFA release from adipocytes. Furthermore, Melon GliSODin^®^ itself would be acts in the same manner as SOD in enhancing the quenching of ROS and reducing oxidative stress.

Overall, our results indicate that Melon GliSODin^®^ prevents NASH by reducing hepatic oxidative stress, which is an intrinsic pathological characteristic of NASH. Our results suggest that SOD-rich Melon GliSODin^®^ serves as a potential antioxidant in the prevention of NASH. 

## Figures and Tables

**Table 1:** Primer sequences.

Gene	Forward Primer (5′→3′)	Reverse Primer (5′→3′)
*18S*	TTCTGGCCAACGGTCTAGACAAC	CCAGTGGTCTTGGTGTGCTGA
*Acc*	ACCCACTCCACTGTTTGTGA	CCTTGGAATTCAGGAGAGGA
*Catalase*	CCAGCGACCAGATGAAGCAG	CCACTCTCTCAGGAATCCGC
*Ccl2*	TTAAAAACCTGGATCGGAACCAA	GCATTAGCTTCAGATTTACGGGT
*Ccr2*	AGCACATGTGGTGAATCCAA	TGCCATCATAAAGGAGCCA
*Ccr2*	AGCACATGTGGTGAATCCAA	TGCCATCATAAAGGAGCCA
*Col1a1*	CCTCAGGGTATTGCTGGACAAC	TTGATCCAGAAGGACCTTGTTTG
*Col3a1*	TTGATGTGCAGCTGGCATTC	GCCACTGGCCTGATCCATAT
*Col4a1*	CACATTTTCCACAGCCAGAG	GTCTGGCTTCTGCTGCTCTT
*Ctgf*	ACCCGAGTTACCAATGACAATACC	CCGCAGAACTTAGCCCTGTATG
*FAS*	TCTGCCAGTGAGTTGAGGAC	CTGCAGAGAAGCGAGCATAC
*GPx1*	AGTCCACCGTGTATGCCTTCT	GAGACGCGACATTCTCAATGA
*IL1b*	CTGAACTCAACTGTGAAATGCCA	AAAGGTTTGGAAGCAGCCCT
*MCP-1*	CCACTCACCTGCTGCTACTCAT	TGGTGATCCTCTTGTAGCTCTCC
*PPARgamma*	TGGCCACCTCTTTGCTCTGCTC	AGGCCGAGAAGGAGAAGCTGTTG
*Resistin*	CCCTCCTTTTCCTTTTCTTCCTTG	AGACTGCTGTGCCTTCTGGG
*Scd1*	CTCCTGCTGATGTGCTTCAT	AAGGTGCTAACGAACAGGCT
*SOD1*	TGAGGTCCTGCACTGGTAC	CAAGCGGTGAACCAGTTGTG
*SOD2*	TTAACGCGCAGATCATGCA	GGTGGCGTTGAGATTGTTCA
*Srebp1c*	CGTGAGCTACCTGGACTGAA	CGGGACAGCTTAGCCTCTAC
*TGF-β*	TTGCTTCAGCTCCACAGAGA	TGGTTGTAGAGGGCAAGGAC
*TIMP*	AGGTGGTCTCGTTGATTTCT	GTAAGGCCTGTAGCTGTGCC
*TNFa*	CTGGGACAGTGACCTGGACT	GCACCTCAGGGAAGAGTCTG

## Abbreviations

NAFLD: non-alcoholic fatty liver disease; NASH, non-alcoholic steatohepatitis; eWAT, epididymal white adipose tissue; FFA, free fatty acids; TG, Triglyceride.
